# Osteogenic Factor Runx2 Marks a Subset of Leptin Receptor-Positive Cells that Sit Atop the Bone Marrow Stromal Cell Hierarchy

**DOI:** 10.1038/s41598-017-05401-1

**Published:** 2017-07-10

**Authors:** Mengyu Yang, Atsushi Arai, Nobuyuki Udagawa, Toru Hiraga, Zhao Lijuan, Susumu Ito, Toshihisa Komori, Takeshi Moriishi, Koichi Matsuo, Kouji Shimoda, Ali H. Zahalka, Yasuhiro Kobayashi, Naoyuki Takahashi, Toshihide Mizoguchi

**Affiliations:** 1 Institute for Oral Science, Matsumoto Dental University, Nagano, 399-0781 Japan; 2 Department of Orthodontics, Matsumoto Dental University, Nagano, 399-0781 Japan; 3 Department of Oral Biochemistry, Matsumoto Dental University, Nagano, 399-0781 Japan; 4 Department of Histology and Cell Biology, Matsumoto Dental University, Nagano, 399-0781 Japan; 5 Division of Instrumental Analysis, Research Center for Human and Environmental Sciences, Shinshu University, Nagano, 390-8621 Japan; 6 Department of Cell Biology, Unit of Basic Sciences, Nagasaki University Graduate School of Biomedical Sciences, Nagasaki, 852-8588 Japan; 7 Laboratory of Cell and Tissue Biology, Keio University School of Medicine, Tokyo, 160-8582 Japan; 8 Laboratory Animal Center, Keio University School of Medicine, Tokyo, 160-8582 Japan; 9 Ruth L. and David S. Gottesman Institute for Stem Cell and Regenerative Medicine Research, Albert Einstein College of Medicine, Bronx, NY 10461 USA; 10 Department of Cell Biology, Albert Einstein College of Medicine, Bronx, NY 10461 USA

## Abstract

Bone marrow mesenchymal stem and progenitor cells (BM-MSPCs) maintain homeostasis of bone tissue by providing osteoblasts. Although several markers have been identified for labeling of MSPCs, these labeled cells still contain non-BM-MSPC populations. Studies have suggested that MSPCs are observed as leptin receptor (LepR)-positive cells, whereas osteoblasts can be classified as positive for Runx2, a master regulator for osteoblastogenesis. Here, we demonstrate, using Runx2-GFP reporter mice, that the LepR-labeled population contains Runx2-GFP^low^ sub-population, which possesses higher fibroblastic colony-forming units (CFUs) and mesensphere capacity, criteria for assessing stem cell activity, than the Runx2-GFP^−^ population. In response to parathyroid hormone (PTH), a bone anabolic hormone, LepR^+^Runx2-GFP^low^ cells increase Runx2 expression and form multilayered structures near the bone surface. Subsequently, the multilayered cells express Osterix and Type I collagen α, resulting in generation of mature osteoblasts. Therefore, our results indicate that Runx2 is weakly expressed in the LepR^+^ population without osteoblastic commitment, and the LepR^+^Runx2-GFP^low^ stromal cells sit atop the BM stromal hierarchy.

## Introduction

Bone marrow (BM) cells belonging to mesenchymal lineages are derived from mesenchymal stem and progenitor cells (MSPCs). BM-MSPCs are traditionally characterized as cells possessing colony forming potential in adherent culture conditions [known as colony-forming unit-fibroblasts, CFU-F] and have the ability to form clonal spheres in nonadherent culture conditions [designated as mesenspheres]^1–3^. The clonally expanded CFU-F colonies and mesenspheres have differentiation potential to osteoblasts, adipocytes and chondrocytes both *in vitro* and *in vivo*. BM-MSPCs can be marked by the expression of leptin receptor (LepR)-Cre, and are distributed nearby blood vessels throughout the whole BM cavity^4–6^. *In vivo* fate mapping approaches demonstrated that LepR^+^ cells differentiate to osteoblasts and adipocytes under normal conditions. The contribution of LepR^+^ cells to chondrocytes is observed during the healing process of bone tissue^5, 6^. There is evidence that LepR-Cre-labeled cells largely overlap with other markers for the BM-MSPC populations including CD31^−^CD45^−^Ter119^−^Nestin-GFP^low^ cells^5, 7^, CXCL12 abundant reticular (CAR) cells^8, 9^, PDGFRβ^+^ cells^5, 6^ and Prx-1-Cre labeled cells^10^. Although these markers make it possible to enrich the BM-MSPCs from whole BM cells, not all the labeled cells have the potential to form CFU-F colonies or clonal mesenspheres^6, 7, 11^. These results suggest that the fractions are impure and still contain non-BM-MSPC populations.

Runt-related transcription factor 2 (Runx2) is a master regulator for osteoblast differentiation^12–14^. Osteoblastogenesis is fully suppressed by the global knockout of Runx2^13, 14^. Exon 8 of Runx2 gene conditional deletion in mature osteoblasts, which express Cre recombinase under the control of a 2.3-kb fragment of the type I collagen α[(Col1(2.3)] promoter, exhibit low bone mass phenotype^15^. In contrast, conditional knockout mice lacking exon 4 of Runx2 gene in mature osteoblasts have no effect on osteoblastic activity^16^. These studies indicate that the necessity of Runx2 in osteoblastic activity is still controversial. On the other hand, i*n vivo* lineage tracing studies have demonstrated that Runx2 is essential for osteoblast lineage commitment^17^. Interestingly, Runx2 overexpression approaches revealed that the late stage of osteoblastogenesis is negatively regulated by Runx2, whose levels were found to decrease with osteoblast maturation^18, 19^. Overall, these findings suggest that Runx2 is required for osteoblast commitment from immature mesenchymal stromal cells. These results raise the intriguing possibility that Runx2 may be expressed in a portion of LepR^+^ stromal cells, which have osteogenic-committed sub-populations.

Osteoblastogenesis is completely diminished in knockout mice lacking Osterix (Osx), a transcription factor that acts downstream of Runx2^20^. Furthermore, bone formation is inhibited by conditionally deleting Osx in mature osteoblasts^21^. These results suggest that Osx is necessary not only for osteoblast differentiation, but also for their functions. On the other hand, during endochondral bone ossification, BM-MSPCs are generated from part of the developing chondrogenic cell populations^17^. The expression levels of Osx are increased throughout the development of chondrogenic cell populations that subsequently differentiate into BM-MSPCs^5, 17, 22^. Although Osx protein expression in BM-MSPCs is completely lost in the adult stage, mRNA expression is maintained^5, 23^. However, the Osx expression pattern during osteoblastogenesis from BM-MSPCs has yet to be elucidated.

Teriparatide, a biologically active amino acid 1–34 fragment of human PTH [hPTH (1–34)], is clinically used in treatment of osteoporosis patients^24^. Several studies have demonstrated that intermittent PTH treatment induces remedial action against osteoporosis due to anabolic effects on bone tissue^25–28^. Researchers have found that osteoblast precursors are increased along the bone surfaces in response to PTH treatment^27–30^. These results suggest that the anabolic effects of PTH on bone tissue are exerted by the acceleration of osteoblastogenesis from immature BM mesenchymal precursors. However, it still remains unclear which BM stromal cells give rise to osteoblasts in response to PTH treatments, thereby mediating the therapeutic response in osteoporosis.

Here we demonstrate, using Runx2-GFP reporter mice, that the LepR^+^ cell population contains Runx2-GFP^low^ cells, and unexpectedly, that stem cell capacity is enriched in the Runx2-GFP^low^ sub-population. In addition, our studies have shown that the LepR^+^Runx2-GFP^low^ cells differentiate into mature osteoblasts via multilayered cell formation adjacent to bone surfaces in response to PTH-induced bone anabolic effects. These results provide evidence that LepR^+^Runx2-GFP^low^ cells sit atop the BM mesenchymal stromal cell hierarchy.

## Results

### Runx2 is heterogeneously expressed in the LepR^+^ BM stromal cell population

**Figure 1:**
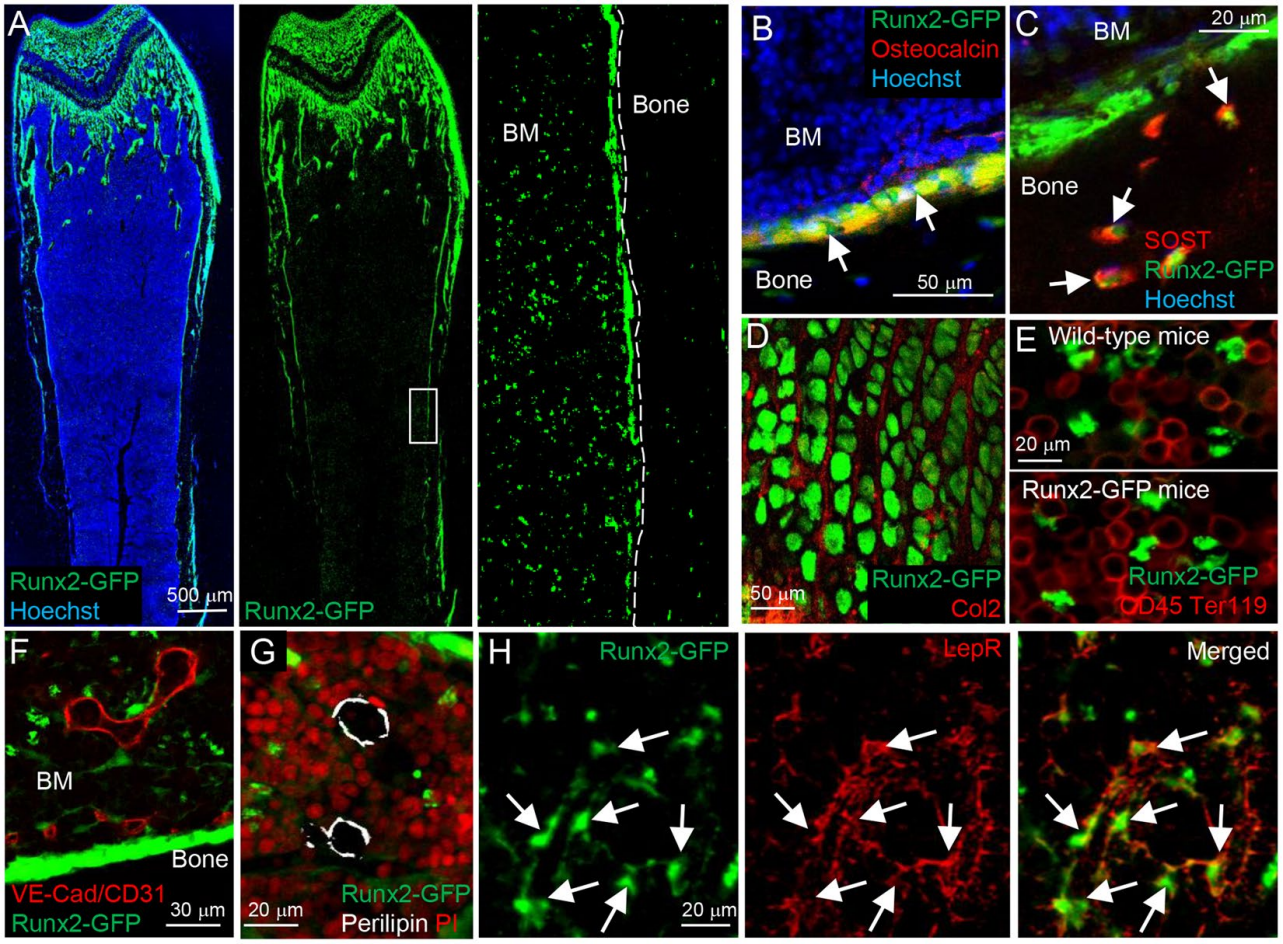
LepR^+^ cells in the bone marrow cavity express Runx2. (**A**–**H**) Z-stack confocal (**A**,**D**–**F** and **H**) and confocal (**B**,**C** and **G**) images of thick bone sections of Runx2-GFP mice (5–6 weeks old). The images were taken from whole bone tissue (**A**), endosteum (**B**,**C** and **F**), epiphyseal cartilage (**D**) and bone marrow (**E**,**G** and **H**). Bone tissues are stained with osteocalcin (Red) (**B**), SOST (Red) (**C**), Type-2 collagen (Col2) (Red) (**D**), CD45 and Ter119 (Red) (**E**), VE-cadherin (VE-Cad) and CD31 (Red) (**F**), Perilipin (White) (**G**), and Leptin receptor (LepR) (Red) (**H**) antibodies. Nuclei are visualized with Hoechst 33342 (blue) and propidium iodide (PI) (Red). Arrows: Osteoblasts (**B**), Osteocytes (**C**), and LepR^+^ cells (**H**).

To detect Runx2 expressing cells in bone tissue, we analyzed Runx2-GFP reporter mice, in which GFP is driven by a bacterial artificial chromosome (BAC) of Runx2 locus^31^. FITC-conjugated anti-GFP antibody was used to amplify the intensity of the GFP signal when imaging bone tissue sections. Consistent with a previous study^31^, Runx2-GFP^+^ expression in bone tissues was detected in osteoblasts, osteocytes and chondrocytes (Fig. 1A–D). Interestingly, the Runx2-GFP signal was observed not only in bone tissues, but also in the BM cavity (Fig. 1A, right panel). Some hematopoietic cells were observed as GFP-positive cells due to nonspecific binding of the anti-GFP antibody (compare wild-type and Runx2-GFP mice in Fig. 1E). On the other hand, VE-cadherin (VE-Cad) and CD31-positive endothelial cells, and Perilipin-positive adipocytes did not express Runx2-GFP (Fig. 1F,G). It is interesting to note that leptin receptor-positive (LepR^+^) BM stromal cells, which are considered to have characteristics of BM-MSPCs, also weakly expressed Runx2-GFP throughout the bone marrow cavity (Fig. 1H). These results indicate that the osteoblastic master regulator Runx2 may already be expressed in the pre- or early osteoblastic lineage-committed LepR^+^ sub-population.

**Figure 2:**
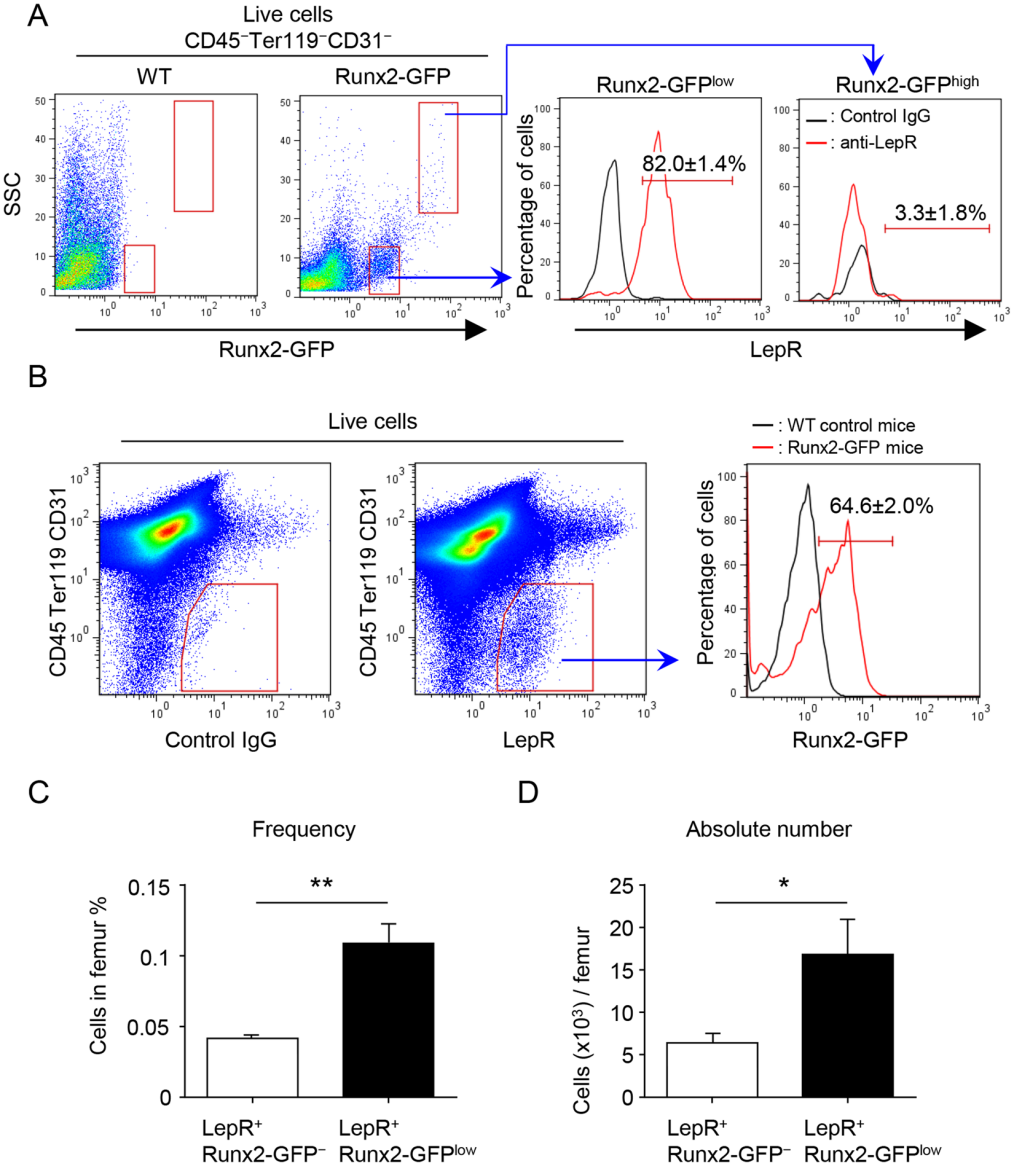
LepR^+^ cells contain Runx2-GFP^low^ and Runx2-GFP^−^ sub-populations. (**A**) Representative FACS plots (gated on live CD45^−^Ter119^−^CD31^−^ cells) showing the expression of LepR in Runx2-GFP^+^ stromal populations from 5 week-old Runx2-GFP mice. Left panel showing representative FACS plot of WT control (gated on live CD45^−^Ter119^−^CD31^−^ cells). Black and red lines represent the isotype control and specific antibody against LepR, respectively. n = 3. (**B**) Representative FACS plots (gated on live cells) showing frequency of Runx2-GFP^+^ population in the CD45^−^Ter119^−^CD31^−^LepR^+^ cell population (right panel) from 5–6 week-old Runx2-GFP mice. Left panel showing negative control for LepR antibody (gated on live cells). Black and red lines represent the WT control and Runx2-GFP mice, respectively (right panel). n = 3. (**C** and **D**) Quantification of the frequency (**C**) and absolute number (**D**) of Runx2-GFP^low^ and Runx2-GFP^−^ sub-populations in LepR^+^ cells (CD45^−^Ter119^−^CD31^−^). n = 3. *P < *0.05*, **P < *0.01*. Data are represented as mean ± SD.

To further analyze Runx2-GFP expression pattern in the BM stromal population, we performed flow cytometric analyses of BM cells in the Runx2-GFP mice. Runx2-GFP^+^ cells were observed in the BM stromal cell population without enhancing the GFP signal by FITC-conjugated anti-GFP antibody (Fig. 2A). We found two distinct types of Runx2-GFP^+^ cells on the basis of their GFP expression levels and cellular morphology (designated as Runx2-GFP^low^ and -GFP^high^ cells) (Fig. 2A). Most of the Runx2-GFP^low^ cells (82.0 ± 1.4%), but not Runx2-GFP^high^ cells (3.3 ± 1.8%), were positive for LepR (Fig. 2A). Previous reports suggested that mature osteoblasts are negative for LepR^5, 6^. These results indicate that the mature osteoblasts are contained in the Runx2-GFP^high^ population. Next, we analyzed the Runx2-GFP expression pattern in LepR^+^ cells. Interestingly, most of the LepR^+^ cells were positive for Runx2-GFP (64.6 ± 2.0%) (designated as LepR^+^Runx2-GFP^low^ cells) (Fig. 2B, right panel). The frequency and absolute number of LepR^+^Runx2-GFP^low^ cells per femur were approximately 3-times higher than those of LepR^+^Runx2-GFP^−^ cells (Fig. 2C,D). These results indicate that LepR^+^ cells consist of two populations: LepR^+^Runx2-GFP^low^ and LepR^+^Runx2-GFP^−^ sub-populations.

### Stem cell activity is enriched in the Runx2-GFP^low^ sub-population of LepR^+^ cells

**Figure 3:**
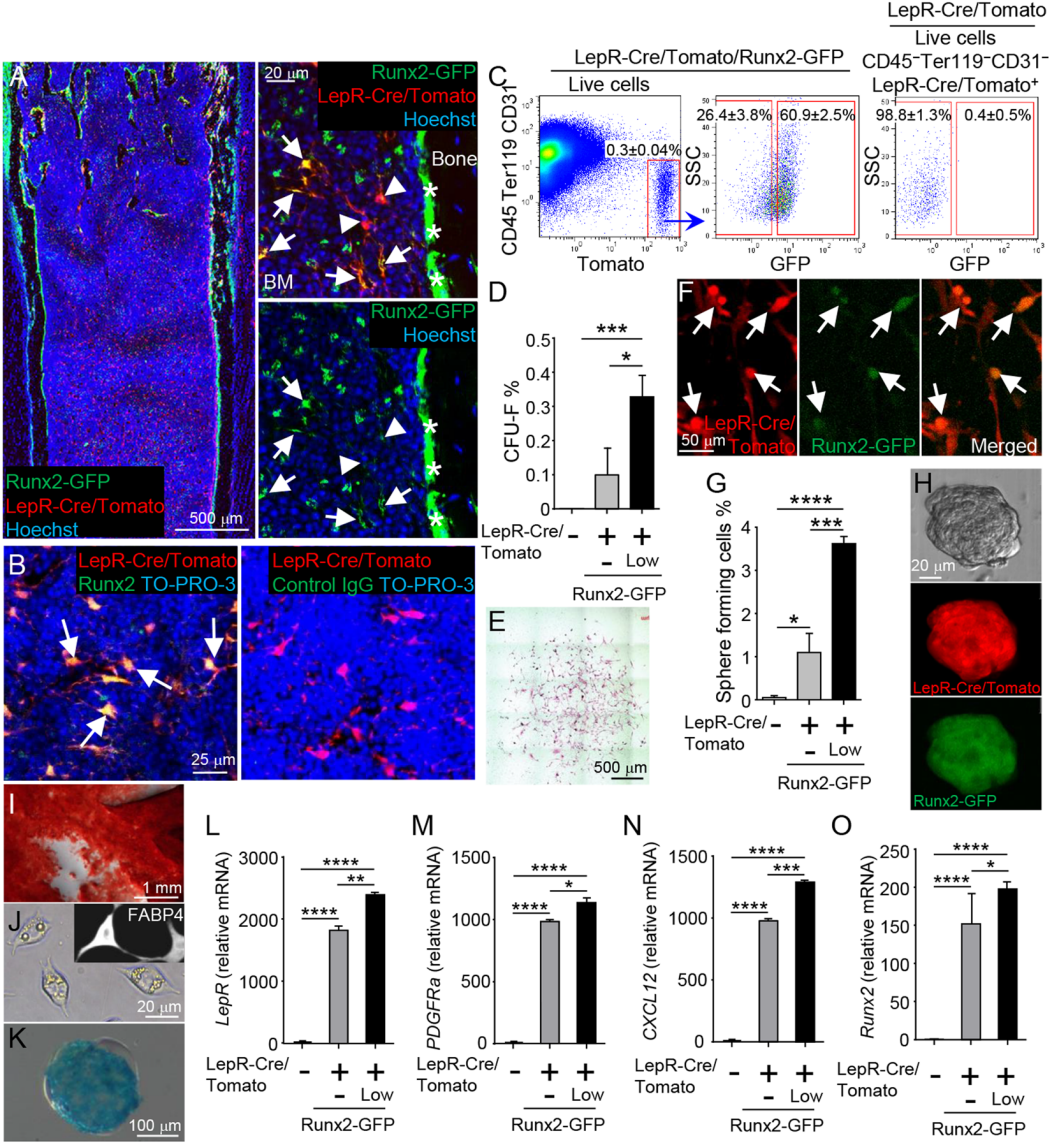
Stromal stem cell activity in BM is enriched in LepR^+^Runx2-GFP^low^ population. (**A**) Z-stack confocal images of thick bone sections of 6 week-old LepR-Cre/Tomato/Runx2-GFP mice. Arrows: LepR-Cre-derived Tomato^+^ (LepR/Tomato^+^)/Runx2-GFP^low^ cells. Arrowheads: LepR/Tomato^+^/Runx2-GFP^−^ cells. *: Runx2-GFP^high^ bone-lining mature osteoblasts. Nuclei were visualized with Hoechst 33342 (blue). (**B**) Z-stack confocal images of thick bone sections of 6 week-old LepR-Cre/Tomato mice stained with Runx2 (left panel, green) and control IgG (right panel). Arrows: LepR/Tomato^+^/Runx2^+^cells. Nuclei are visualized with To-PRO-3 (blue). (**C**) Representative FACS plots (gated on live cells) showing the percentages of Runx2-GFP-positive (designated as Runx2-GFP^low^) and -negative (designated as Runx2-GFP^−^)cells (middle panel) in the CD45^−^Ter119^−^CD31^−^LepR/Tomato^+^ stromal population (left panel) from 6 week-old LepR-Cre/Tomato/Runx2-GFP mice. Right panel showing representative FACS plot of control (gated on live CD45^−^Ter119^−^CD31^−^LepR/Tomato^+^ cells) in 6 week-old LepR-Cre/Tomato mice. (**D**–**O**) CD45^−^Ter119^−^CD31^−^ stromal cells (gated on live cells) were sorted based on expression of LepR-Cre/Tomato and Runx2-GFP from 6-7 week-old LepR-Cre/Tomato/Runx2-GFP mice, and percentage of CFU-F (**D**) and clonal sphere (mesensphere) formation (**G**) were determined. Representative image of CFU-F colony (**E**; Giemsa staining, **F**; Tomato and GFP fluorescence). n = 3 independent experiments. Arrows: LepR/Tomato and Runx2-GFP double-positive cells. Representative image of mesensphere formation (**H**; bright field, Tomato, and GFP fluorescence). n = 3 independent experiments. Differentiation phenotypes of LepR/Tomato^+^/Runx2-GFP^low^ cells shown by Alizarin Red S: osteoblasts (**I**), lipid droplets and staining with FABP4 antibody: adipocytes (**J**), and Alcian Blue: chondrocytes (**K**). Expression levels of *LepR* (**L**), *PDGFRa* (**M**), *CXCL12* (**N**) and *Runx2* (**O**) were measured by quantitative real-time PCR. n = 3–5. *P < 0.05, **P < *0.01*, ***P < 0.001, ****P < *0.0001*. Data are represented as mean ± SD.

Upregulation of Runx2 expression is thought to indicate osteoblastic commitment of multipotent BM stromal progenitors^32^. We next generated LepR-Cre/ROSA26-loxP-stop-loxP-tdTomato/Runx2-GFP (LepR/Tomato/Runx2-GFP) mice, and analyzed whether stem cell activity in LepR^+^ cells increases in inverse proportion to Runx2 expression levels. Previous reports demonstrated that the differentiation of LepR^+^ cells into mature osteoblasts is mainly observed in adult mice, and only rarely seen in young mice^5, 6^. In order to avoid contamination of the LepR-Cre-labeled population with LepR^+^ cell-derived osteoblasts, we analyzed young mice (5–7 weeks old) in this experiment. Histological analysis demonstrated that bone-lining osteoblasts were strongly positive for Runx2-GFP, but negative for LepR/Tomato (Fig. 3A, asterisks). These results indicate that LepR-Cre-labeled Tomato^+^ (LepR/Tomato^+^) cells do not contain the osteoblastic population in young mice. On the other hand, the LepR/Tomato^+^ cells in the BM cavity were positive for Runx2-GFP (Fig. 3A, arrows). Immunofluorescence staining showed that LepR/Tomato^+^ cells express Runx2 protein (Fig. 3B, arrows). Flow cytometric analysis also demonstrated that the majority of LepR/Tomato^+^ cells in BM expressed Runx2-GFP (60.9 ± 2.5%) (Fig. 3C). We then assessed the stem cell activity of both Runx2-GFP^low^ and Runx2-GFP^−^ sub-populations in LepR/Tomato^+^ cells by performing CFU-F assays of cell sorted BM stromal fractions (Fig. 3D). Consistent with previous reports^6, 7^, there were no CFU-F capable cells in the LepR/Tomato^−^ BM stromal population (Fig. 3D). Contrary to our expectations, the CFU-F capacity of the LepR/Tomato^+^/Runx2-GFP^low^ BM stromal population was high (Fig. 3D). CFU-F colonies derived from the LepR/Tomato^+^/Runx2-GFP^low^ BM stromal population were positive for LepR/Tomato and weakly Runx2-GFP-positive (Fig. 3E,F). Furthermore, when BM stromal fractions were plated at clonal densities under nonadherent culture conditions, the LepR/Tomato^+^/Runx2-GFP^low^ population formed spheres (mesenspheres) at greater frequency than other fractions (Fig. 3G,H). The LepR/Tomato^+^/Runx2-GFP^low^ BM stromal cells exhibited tri-lineage differentiation potential (Fig. 3I–K). However, it is not clear whether the LepR/Tomato^+^/Runx2-GFP^low^ BM stromal cells generate other types of BM stromal cells. Real-time PCR analyses revealed that the LepR/Tomato^−^ sub-population did not express the MSPC markers *PDGFRa* ^7, 33^ *LepR* ^5, 6^ or *CXCL12* ^23^(Fig. 3L–N). Interestingly, the expression levels of all three of these MSPC markers in the LepR-Cre/Tomato^+^Runx2-GFP^low^ sub-population were significantly higher than in the LepR-Cre/Tomato^+^Runx2-GFP^−^ sub-population (Fig. 3L–N). These results also indicated that the LepR-Cre/Tomato^+^Runx2-GFP^low^ sub-population overlaps with CXCL12 abundant reticular (CAR) cells^23^, which are generated from part of the developing chondrogenic cell populations^17^. The expression levels of *Runx2* mRNA in the Runx2-GFP^low^ sub-population were significantly higher than those in the Runx2-GFP^−^ sub-population from LepR-Cre/Tomato^+^ stromal cells (Fig. 3O). The *Runx2* expression was hardly detected in the LepR-Cre/Tomato^−^ stromal cell population at the mRNA level (Fig. 3O). Consistently, the LepR-Cre/Tomato^−^ stromal population contained almost no Runx2-GFP^+^ cells (0.7 ± 0.2%) (Suppl. Fig. 1). Taken together, these results indicate that stromal stem cell activity in BM is high in LepR^+^ Runx2-GFP^low^ stromal cell populations. Therefore, our findings provide evidence that LepR^+^ Runx2-GFP^low^ cells sit atop the BM stromal cell hierarchy, and the osteoblastic master transcription factor Runx2 is weakly expressed in BM-MSPC populations without osteoblastic lineage commitment.

### LepR^+^Runx2-GFP^low^ cells differentiate into osteoblasts through multilayered cell formation in response to PTH anabolic effects

**Figure 4:**
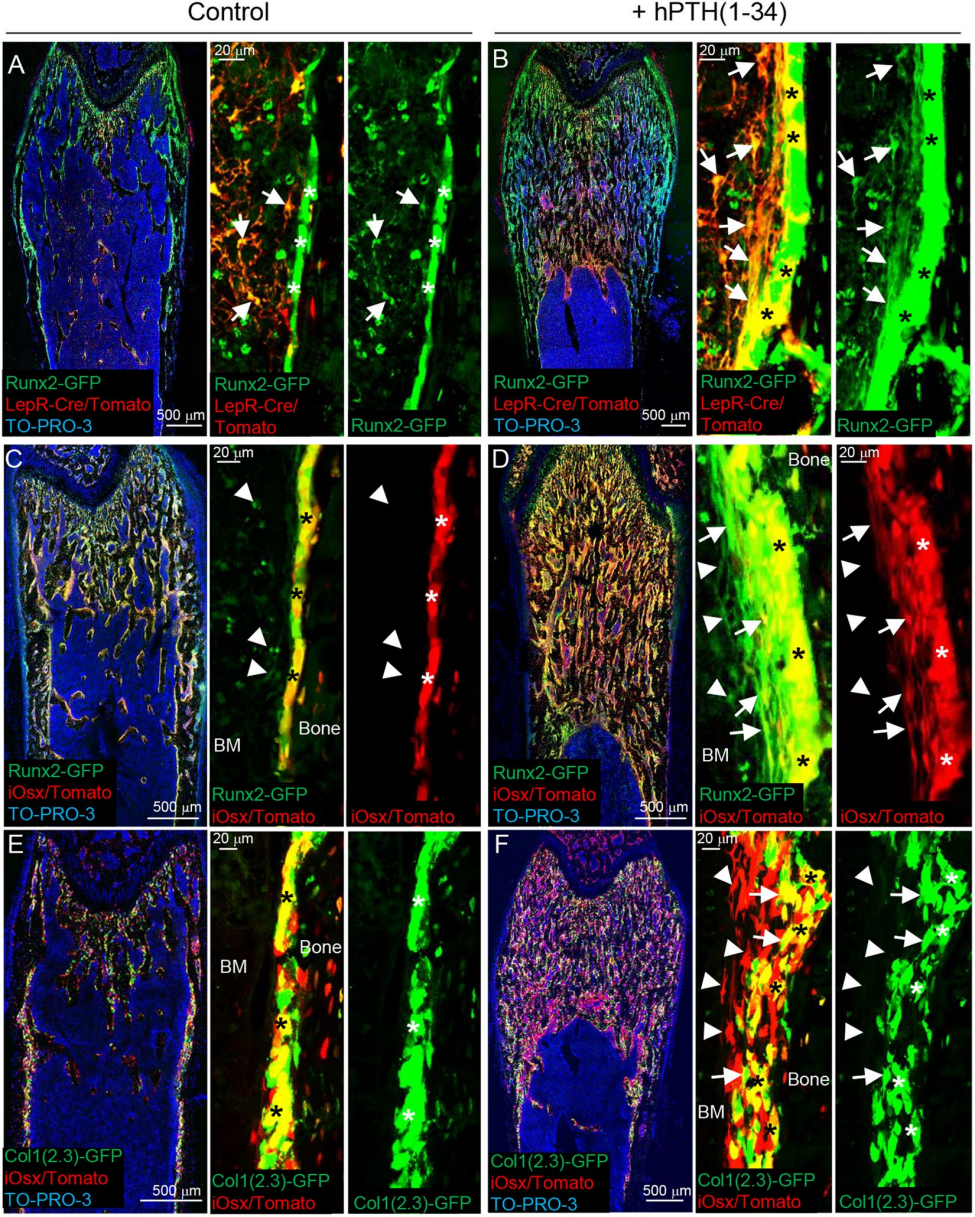
LepR^+^Runx2-GFP^low^ cells differentiate into osteoblasts through multilayered cell formation in response to PTH-induced anabolic effects. (**A**–**F**) Z-stack confocal images of thick bone sections of 6 week-old LepR-Cre/Tomato/Runx2-GFP mice (**A** and **B**), tamoxifen-administered iOsx/Tomato/Runx2-GFP mice (**C** and **D**), and tamoxifen-administered iOsx/Tomato/Col1(2.3)-GFP mice (**E** and **F**) with vehicle (**A**,**C** and **E**) and hPTH(1–34) (**B**,**D** and **F**) intermittent treatment. Arrows: LepR-Cre-derived Tomato^+^(LepR/Tomato^+^)Runx2-GFP^+^ cells (**A** and **B**), iOsx/Tomato^+^Runx2-GFP^+^ cells (**D**) and iOsx/Tomato^+^Col1(2.3)-GFP^+^ cells (**F**). Arrowheads: iOsx/Tomato^−^Runx2-GFP^+^ cells (**C** and **D**) and iOsx/Tomato^+^Col1(2.3)-GFP^−^ cells (**F**). *: Bone-lining mature osteoblasts. Nuclei were visualized with To-PRO-3 (blue).

*In vivo* genetic lineage tracing analysis demonstrated that LepR^+^ cells differentiate into osteoblasts^5, 6^. As we found that stem cell activity is enriched in LepR^+^Runx2-GFP^low^ BM stromal cell populations, we next examined whether LepR^+^Runx2-GFP^low^ cells differentiate into mature osteoblasts *in vivo* by lineage tracing. Because intermittent treatment of parathyroid hormone (PTH) (1–34) increased bone volume by inducing bone formation (Suppl. Fig. 2)^24–26, 28^, we injected PTH into LepR/Tomato/Runx2-GFP mice. Bone-lining osteoblasts were detected as Runx2-GFP single-positive cells in the control group (Fig. 4A, asterisks). In contrast, LepR/Tomato and Runx2-GFP double-positive mature osteoblasts were significantly increased on the endosteal surface in the PTH-treated bone tissue (Fig. 4B, asterisks and Suppl. Fig. 3A). These results suggest that osteoblastogenesis from LepR^+^ cells is accelerated by PTH treatment. It is noteworthy that while LepR^+^Runx2-GFP^low^ cells were observed in the control BM cavity (Fig. 4A and Suppl. Fig. 4A, arrows), LepR^+^Runx2-GFP^+^ multilayered cells (designated as ML-cells) were observed in the vicinity of bone tissue by PTH treatment (Fig. 4B and Suppl. Fig. 4B, arrows). Quantification of vertical- and cross-section images revealed that ML-cells were significantly increased by PTH treatment (Suppl. Figs 3B and 4C–E). The expression level of Runx2-GFP in ML-cells was higher than in LepR^+^Runx2-GFP^low^ cells (Fig. 4A,B, right panels, arrows). In contrast, the expression level of Runx2-GFP in ML-cells was lower than in cuboidal-shaped osteoblasts (Fig. 4B, right panel, arrows and asterisks). These results suggest that the LepR^+^Runx2-GFP^low^ cells differentiate into mature osteoblasts through ML-cell formation with increasing levels of Runx2 expression.

### LepR^+^Runx2-GFP^low^ cell-derived multilayered cells differentiate into mature osteoblasts with increasing expression of Osterix and type I collagen α

We next analyzed the expression pattern of Osterix (Osx), a transcription factor downstream of Runx2, in ML-cells using Osx-Cre^ERT2^(iOsx)/Tomato/Runx2-GFP mice, administering tamoxifen for labeling of Osx^+^ cells^34^. Histological analyses demonstrated that mature osteoblasts express both iOsx/Tomato and Runx2-GFP, but Runx2-GFP^low^ cells located away from bone surfaces were negative for iOsx/Tomato (Fig. 4C, asterisks and arrowheads). ML-cells located far from osteoblasts were iOsx/Tomato negative, but those near bone-lining osteoblasts were positive for Tomato (Fig. 4D, arrowheads and arrows). Both the iOsx/Tomato-negative and -positive ML-cells (Runx2-GFP^+^ cells) were significantly increased by the PTH treatment (Suppl. Fig. 3C and D).

**Figure 5:**
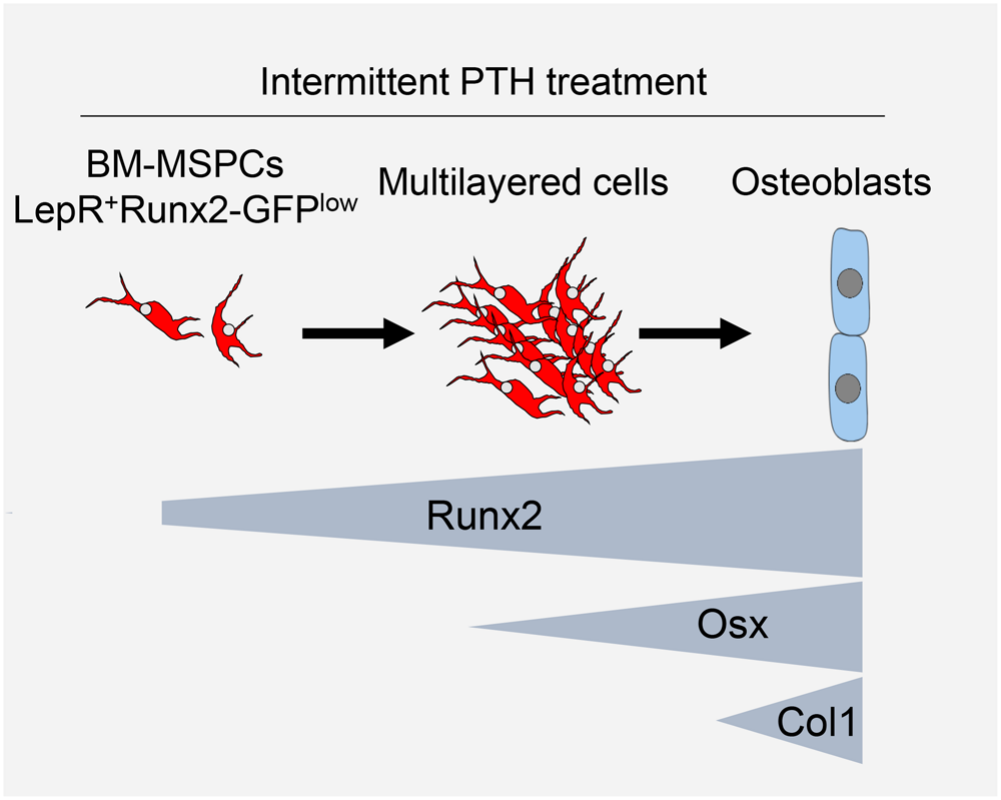
Differentiation model of LepR^+^Runx2-GFP^low^ cells into osteoblasts. LepR^+^Runx2-GFP^low^ cells form multilayered structures along the bone surface in response to intermittent PTH treatment. In this process, the LepR^+^Ranx2-GFP^low^ cells increase the expression levels of Runx2, Osx and Col1 sequentially, and eventually differentiate into mature osteoblasts.

Lastly, we examined the hierarchical relationship between Osx and Type-1 collagen α (Col1), a marker for mature osteoblasts, in osteoblastogenesis from ML-cells using iOsx/Tomato/Col1(2.3)-GFP mice. Tamoxifen-induced iOsx/Tomato^+^ cells were observed as mature osteoblasts with Col1(2.3)-GFP expression in control bone tissue (Fig. 4E, asterisks). However, iOsx/Tomato^+^ cells were observed as ML-cells after PTH-treatment (Fig. 4F). Only the population of these cells localized in the vicinity of the bone surface overlapped with Col1(2.3)-GFP expression (Fig. 4F, compare arrowheads and arrows). Both the Col1(2.3)-GFP-negative and -positive ML-cells (iOsx/Tomato^+^ cells) were significantly increased by the PTH treatment (Suppl. Fig. 3E and F). These results thus suggest that LepR^+^Runx2-GFP^low^ cells differentiate into ML-cells adjacent to the bone surface, and that PTH treatment enhances Runx2 expression, which subsequently induces Osx expression, resulting in differentiation into Col1^+^ mature osteoblasts (Fig. 5).

## Discussion

To clarify *in vivo* osteoblastogenesis from LepR^+^ cells, we analyzed the expression pattern of the early osteoblastic transcription factor, Runx2, in LepR^+^ cells using Runx2-GFP transgenic mice^31^. Here we report that BM-MSPCs in adult BM are confined to the weak Runx2-GFP-expressing LepR^+^ stromal cell population, which differentiates into Col1^+^ mature osteoblasts in response to PTH anabolic effects. In this process, the LepR^+^Runx2-GFP^low^ cells form multilayered structures along the bone surface, subsequently increasing expression levels of Runx2 and Osx.

Our results demonstrating that the stem cell capacity is enriched in Runx2-GFP^low^ populations are consistent with recently published data in which the most primitive stromal population in the calvaria, gated as Prx1^+^Scal^+^ cells, expresses Runx2 at low levels^35^. On the other hand, a single-cell assay demonstrated that CXCL12 abundant reticular (CAR) cells, which largely overlap with the LepR^+^ cell population, express not only Runx2 and Osx, but also peroxisome proliferator-activated receptor γ (PPARγ), an essential transcription factor for adipogenesis^36^, at the mRNA level^23^. Interestingly, osteoblastogenesis is enhanced by decreased transcriptional activity and haploinsufficiency of PPARγ in BM stromal progenitors^37, 38^. In contrast, adipogenesis is accelerated due to the stromal deletion of Wnt/β-catenin-signaling, an essential signaling pathway for osteoblastogenesis^39^. These reports suggest that the undifferentiated state of LepR^+^Runx2-GFP^low^ cells is sustained due to reciprocal inhibition between osteogenic and adipogenic factors. Several studies have reported that the lineage differentiation of BM-MSPCs is skewed toward osteoblasts by intercellular expression of vascular endothelial growth factor A (VEGF-A)^40^. In contrast, the adipocyte lineage commitment from BM-MSPCs is increased by up-regulation of MicroRNA-188^41^, deletion of transcription factor Foxc1^9^ and peripheral Leptin/Leptin receptor signaling^10^. Further studies will clarify the mechanistic details of the cell fate decision of BM-MSPCs.

Our data demonstrate that CFU-F and mesensphere forming capacities are rarely observed in the Runx2-GFP^−^ stromal sub-population of LepR^+^ cells. These results indicate that the LepR^+^Runx2-GFP^−^ stromal sub-population contains some kind of committed cells other than stem cells. Previous studies provide *in vivo* evidence demonstrating that LepR^+^ cells differentiate into not only osteoblasts but also adipocytes with aging and during tissue regeneration processes after injury^5, 6^. In the process of adipogenesis, the expression levels of Runx2 decrease, as opposed to the increase of PPARγ^40^. Furthermore, our immunofluorescence data demonstrated that adipocytes are negative for Runx2 (Fig. 1G). These data suggest that the LepR^+^Runx2-GFP^−^ stromal sub-population may be adipocyte-committed precursors. Further analysis of this fraction will provide information about the process of adipocytogenesis from BM-MSPCs *in vivo*.

In this study, we demonstrated that the PTH treatment accelerated osteoblastogenesis from LepR^+^Runx2-GFP^low^ cells. It has been shown that mature osteoblasts are continuously replaced by immature precursors in adult bone tissues^42^. Because the LepR^+^ cells differentiate into osteoblasts in adult bone tissues^5, 6^, the LepR^+^Runx2-GFP^low^ cells contribute to bone remodeling and maintain bone homeostasis in the adult phase. On the other hand, it has been reported that PTH anabolic effects are exerted by activation of quiescent bone lining osteoblasts^43^, suggesting that the PTH has multiple targets for bone anabolism. Our results also demonstrate that ML-cells appear along the bone surface by PTH treatment. Consistent with our results, it is reported that clustered Osx-positive cells are observed as pre-osteoblasts in the vicinity of trabecular bone surfaces from PTH-treated rats^27^. Others, by employing proliferating cell labeling experiments, have also reported that the PTH-induced thick layered cells proliferate with expression of osteoblastic markers such as alkaline phosphatase (ALP), Runx2, osteocalcin and osteonectin^29, 30^. These results indicate that the PTH-induced osteoblastic differentiation is associated with cell cycle progression. Previous studies reported that the LepR^+^ cells are quiescent in adult BM^5, 6^. However, the LepR^+^ cells markedly proliferate in response to self-depletion, and lineage differentiation into both osteoblasts and adipocytes is accelerated concomitantly in this situation^6^. In contrast, depletion of the transcription factors Snail and Slug in the skeletal stem cells decreases not only proliferative activity but also lineage commitment potential, coincidently^44^. These results suggest that cell cycle quiescence may be critical for maintaining the undifferentiated state of BM-MSPCs. Further analysis of the mechanistic relationship between cell cycle regulation and lineage commitment of LepR^+^Runx2-GFP^low^ cells will provide a potential therapeutic target for osteoporotic patients.

## Experimental Procedures

### Experimental Animals

C57BL/6, B6.129-Lepr*tm2(cre)Rck*/J (LepR-*Cre*), B6.Cg-*Gt(ROSA)26Sortm14(CAG-tdTomato)Hze*/J mice were purchased from Jackson Laboratory. Osx-*Cre* ^*ERT2*^ mice^34^ were provided from H.M. Kronenberg. Col1(2.3)-*Gfp* mice^45^ and Runx2-*Gfp* mice^31^ were generated in one of the author’s laboratories, and backcrossed with C57BL/6 for 5 generations. 5–7 week-old mice were used for all experiments. All mice were maintained in pathogen-free conditions in animal facilities certified by the Animal Care and Use Committees of Matsumoto Dental University, and animal protocols were approved by that committee. All animal studies were performed in accordance with the Guidelines of the Matsumoto Dental University Animal Care Committee.

### Antibodies and reagents

The primary antibodies used were Alexa Fluor 647-anti-VE-Cadherin and Alexa Fluor 647-anti-CD31/PECAM-1 (MEC13.3) (all from Biolegend); APC or PE-anti-CD45 (30-F11), APC or PE-anti-Ter119 (Ter119) (all from eBioscience); anti-LepR, anti-SOST/Sclerostin and anti-fatty acid binding protein 4 (FABP4) (all from R&D systems); anti-Osteocalcin (R21C-01A) and anti-DMP-1 (all from TAKARA); anti-Perilipin (Novus Biologicals); anti-chick typeII collagen (A2-10) (Chondrex); anti-Runx2 (D1L7F) (Cell Signaling). The secondary antibodies used were Alexa Fluor 647 donkey anti-goat IgG and Alexa Fluor 594 donkey anti-rat IgG (all from Molecular probes); Cy3 donkey anti-mouse IgG (Merck Millipore); FITC donkey anti-rabbit IgG (Bethyl Laboratories). Alexa Fluor 488-anti-GFP (Molecular Probes) was used for enhancement of the Runx2-GFP signal. Nuclei were stained with Hoechst 33342 (Sigma-Aldrich) or TO-PRO-3 Iodide (642/661) (Molecular Probes).

### PTH treatment and induction of Cre-mediated recombination

Human PTH (1-34) was kindly provided from Asahi Kasei Pharma Co. Ltd. Four-six-week-old mice were intraperitoneally injected with PTH (80 μg/kg/12 hours) for 10 days. Forty-eight hours after the final PTH injection, mice were sacrificed and used for analyses. For induction of Cre-mediated recombination in Osx-Cre^ERT2^ mice, CRF-1 chow diet (Oriental Yeast) containing tamoxifen (Sigma-Aldrich) at 400 mg/kg was given from 5 days before the first PTH injection until the end of the experiment.

### Microscopy imaging

Mice were perfused with 4% paraformaldehyde (PFA) for fixation, and bone tissue were further fixed with 4% PFA for 24 hours at 4 °C, and incubated in 10%, 20% and 30% sucrose each for 2 ho﻿urs at 4 °C for cryoprotection, then embedded in 5% carboxymethyl cellulose (SECTION-LAB). Sections, 10–20-μm thick, were prepared using Kawamoto’s film method^46^. Z-stack confocal projection images were obtained from 2-μm interval images from 10–20-μm thick sections. Fluorescence and phase-contrast images were acquired using a laser-scanning confocal microscope (LSM510, Carl Zeiss) equipped with Plan-Apochromat (10×/0.45 and 20×/0.8), ZEN and Axiovision software (Carl Zeiss). Bright-field images were acquired using a Light microscope Zeiss Axiovert 200 (Carl Zeiss) equipped with Plan-NEOFLUAR (2.5×/0.075), LD A-plan (40×/0.50 Ph2) and Axiovision software (Carl Zeiss) and Stemi 2000-C (Carl Zeiss).

### Preparation of BM cell suspension

BM was gently flushed in L-15 FACS buffer^47^. BM was digested with 0.1% collagenase IV (Gibco), 0.2% Dispase (Gibco) and 20 U/ml DNase (Worthington Biochemical) in HBSS (Gibco) for 30 min at 37 °C.

### CFU-F assay

Mouse sorted cells were seeded at 2–3 × 10^3^ cells per well in a 12-well adherent tissue culture plate using a MesenCult proliferation Kit with MesenPure (StemCell Technologies) containing 100 U/ml and 100 μg/ml penicillin-streptomycin. Half of the media was replaced after 7 days and at day 10, cells were stained with Giemsa staining solution (EMD Chemicals) and adherent colonies were counted.

### Spheroid formation assay

Mouse sorted cells at 1 × 10^3^ were transferred to non-adherent 24 well plates (Corning) with spheroid-forming media^5, 7, 47^ [1:2 ratio of DMEM F12 (Gibco) and Human Endothelial Medium (Gibco) supplemented with 3.75% Chicken Extract (US Biological), 0.1 mM β-ME (Invitrogen), 1% Non-essential amino acids (Gibco), 1% Pen-strep (Gibco), 1% N2 (Gibco), 2% B27 (Gibco), 20 ng/mL human bFGF (R&D Systems), 20 ng/mL mouse PDGF (Peprotech), 20 ng/mL mouse oncostatin M (R&D Systems), 20 ng/mL mouse IGF-1 (Peprotech), 20 ng/mL mouse EGF (Peprotech)]. After 7 days, the spheroid efficiency was determined.

### Cell sorting and flow cytometry

Cell sorting experiments were performed using an Aria III Cell Sorter (BD Biosciences). Flow cytometric analyses were carried out using a Cytomics FC 500 flow cytometer equipped with CXP software (all Beckman Coulter Life Sciences). Dead cells and debris were excluded by FSC, SSC, DAPI (Dojindo) and Fixable Viability Dye eFluor 780 (eBioscience) staining profiles. Data were analysed with FlowJo (Tree Star) software.

### *In vitro* cell differentiation

Sorted LepR-Cre/Tomato^+^Runx2-GFP^low^ stromal cells were expanded using a MesenCult proliferation Kit with MesenPure (StemCell Technologies) containing 100 U/ml and 100 μg/ml penicillin-streptomycin. Osteogenic, adipogenic and chondrogenic differentiation were induced using a Mouse Mesenchymal Stem Cell Function Identification Kit (R&D Systems). Cells were maintained with 5% CO_2_ in a water-jacketed incubator at 37 °C for 2–5 weeks. Mineralized osteogenic cells were identified by Alizarin Red S (Sigma-Aldrich) staining. Adipocytes were identified by characteristic production of lipid droplets and staining with an anti-FABP antibody (R&D Systems). Chondrocytic cells were identified using an Alcian Blue 8GX solution (Sigma-Aldrich).

### RNA isolation and quantitative real-time PCR

Sorted cells were collected in TRIzol reagent (Ambion) and mRNA was purified using a PureLink RNA Micro kit (Invitrogen). Reverse transcription and quantitative real-time PCR were performed using a One Step SYBR Prime Script PLUS RT-PCR kit (TAKARA) and an Applied Biosystems StepOnePlus^TM^ (Applied Biosystems). Gene expression data was normalized to *Gapdh*. The sequences of primers for each gene were as follows: *Gapdh*, 5′-TGTGTCCGTCGTGGATCTGA-3′ (forward) and 5′-TTGCTGTTGAAGTCGCAGGAG-3′ (reverse); *Runx2* (Type II isoform), 5′-CCAGCCACCGAGACCAACC-3′ (forward) and 5′-GTTTGACGCCATAGTCCCTCC-3′ (reverse); *LepR*, 5′-TCAGAATTTTGGGTGGAAAA-3′ (forward) and 5′-GTCCAGGTGAGGAGCAAGAG-3′ (reverse); *PDGFRa*, 5′-AGCAAACATCTTGACTTGGGAACA-3′ (forward) and 5′-ACTTGCATCATTCCCGGACAC-3′ (reverse); *CXCL12*, 5′-CCAGAGCCAACGTCAAGCAT-3′ (forward) 5′-CAGCCGTGCAACAATCTGAA-3′ (reverse).

### Microcomputed tomography analysis

Femora were fixed in 70% ethanol. Three-dimensional (3D) reconstructions of distal femora were obtained by micro-computed tomography (μCT) (ScanXmate-A080, Comscan Tecno). Morphological indices were calculated in trabecular bones located from 0.5 to 1.5 mm from the growth plates using image analysis software (TRI/3D-BON, Ratoc Syatem Engineering).

### Statistics

The results were expressed as mean ± SD. Data were evaluated by unpaired Student′s *t*-tests. Experiments were performed three times and similar results were obtained. Statistical analyses were performed with Graph Pad Prism 6. *P* < 0.05 was considered significant.

## Electronic supplementary material

Supplementary Information
