# Supplementary material for: Osteogenic Factor Runx2 Marks a Subset of Leptin Receptor-Positive Cells that Sit Atop the Bone Marrow Stromal Cell Hierarchy
Source: Sci Rep. 2017 Jul 10;7:4928. doi: 10.1038/s41598-017-05401-1 (PMC5503992; doi:10.1038/s41598-017-05401-1)
Supplement: Supplementary file 1 — Supplementary Information [file 41598_2017_5401_MOESM1_ESM.doc]

Supplementary Information

Osteogenic Factor Runx2 Marks a Subset of Leptin Receptor-Positive Cells that Sit Atop the Bone Marrow Stromal Cell Hierarchy

Mengyu Yang^1, *^, Atsushi Arai^2, *^, Nobuyuki Udagawa^3^, Toru Hiraga^4^, Zhao Lijuan^1^, Susumu Ito^5^, Toshihisa Komori^6^, Takeshi Moriishi^6^, Koichi Matsuo^7^, Kouji Shimoda^8^, Ali Zahalka^9,10^, Yasuhiro Kobayashi^1^, Naoyuki Takahashi^1^, Toshihide Mizoguchi^1^

^1^Institute for Oral Science, ^2^Department of Orthodontics, ^3^Department of Oral Biochemistry, ^4^Department of Histology and Cell Biology, Matsumoto Dental University, Nagano 399-0781, Japan. ^5^Division of Instrumental Analysis, Research Center for Human and Environmental Sciences, Shinshu University, Nagano 390-8621, Japan ^6^Department of Cell Biology, Unit of Basic Sciences, Nagasaki University Graduate School of Biomedical Sciences, Nagasaki 852-8588, Japan. ^7^Laboratory of Cell and Tissue Biology, ^8^Laboratory Animal Center, Keio University School of Medicine, Tokyo 160-8582, Japan. ^9^Ruth L. and David S. Gottesman Institute for Stem Cell and Regenerative Medicine Research, ^10^Department of Cell Biology, Albert Einstein College of Medicine, Bronx, NY 10461, USA.

*These authors contributed equally to this work

Correspondence: [toshim@po.mdu.ac.jp](mailto:toshim@po.mdu.ac.jp); Phone: +81 263-51-2233; Fax: +81 263-51-2223

**
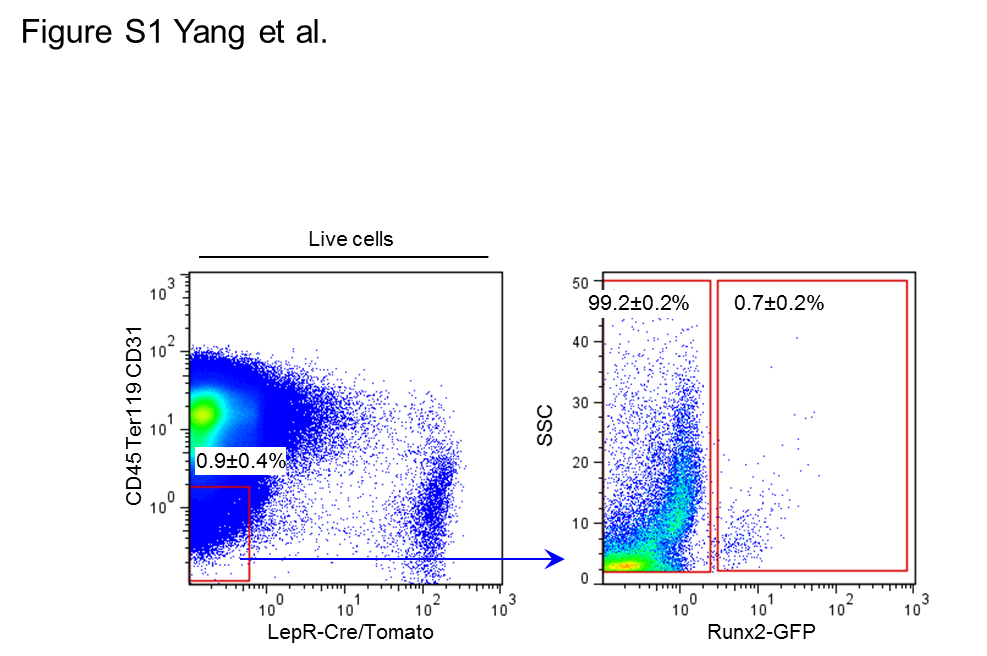
**

**Figure S1. Runx2-GFP^+^ cells are hardly detected in the CD45^−^Ter119^−^CD31^−^LepR/Tomato^−^ stromal population**

Representative FACS plots (gated on live cells) showing the percentages for Runx2-GFP-positive and -negative cells (right panel) in the CD45^−^Ter119^−^CD31^−^LepR/Tomato^−^ stromal population (left panel) from 6 week-old LepR-Cre/Tomato/Runx2-GFP mice.


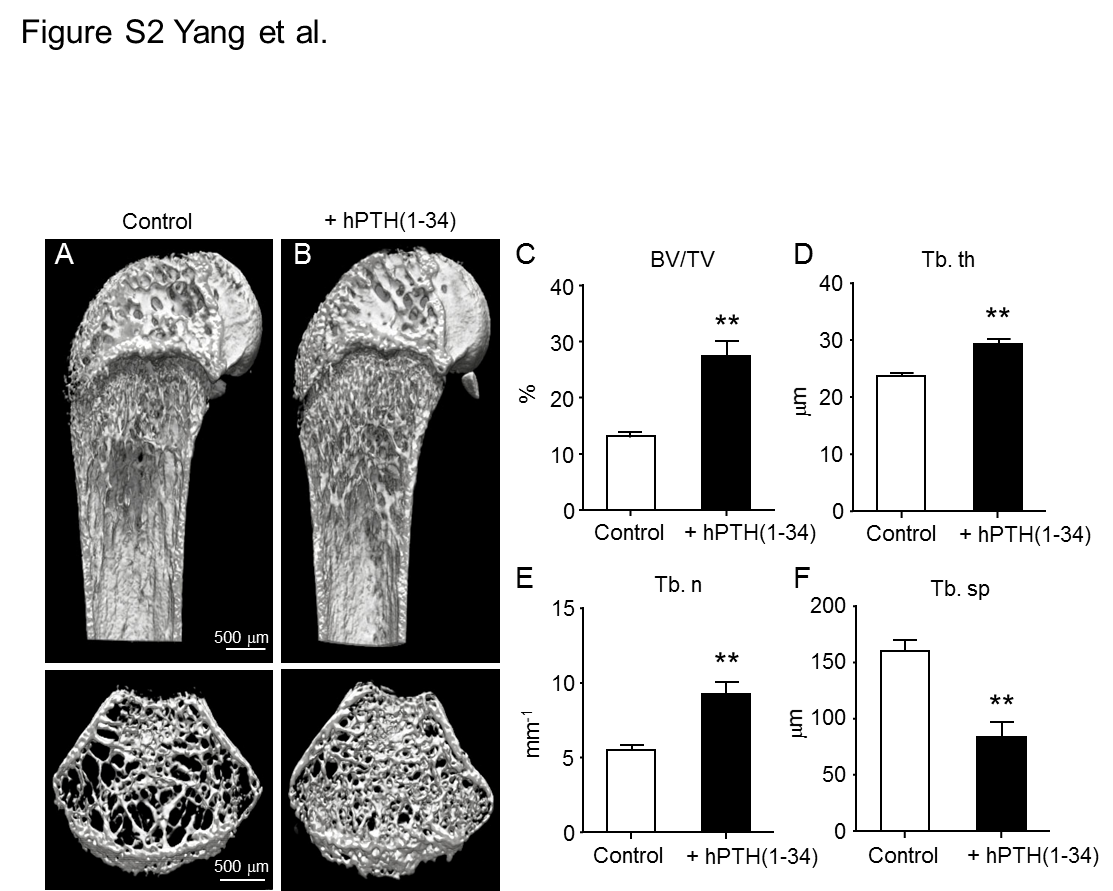


**Figure S2. Intermittent PTH treatment increases bone mass of wild-type mice**

Six week-old wild-type mice were treated with vehicle and hPTH(1-34). Representative micro-CT images (A and B) and quantification of bone parameters (C-F). BV/TV, trabecular bone volume/total volume ratio (C), Tb. th., trabecular thickness (D); Tb. n., trabecular number (E); Tb. sp., trabecular separation (F). n=6. ***P<0.01*. Data are represented as mean ± SD.

**
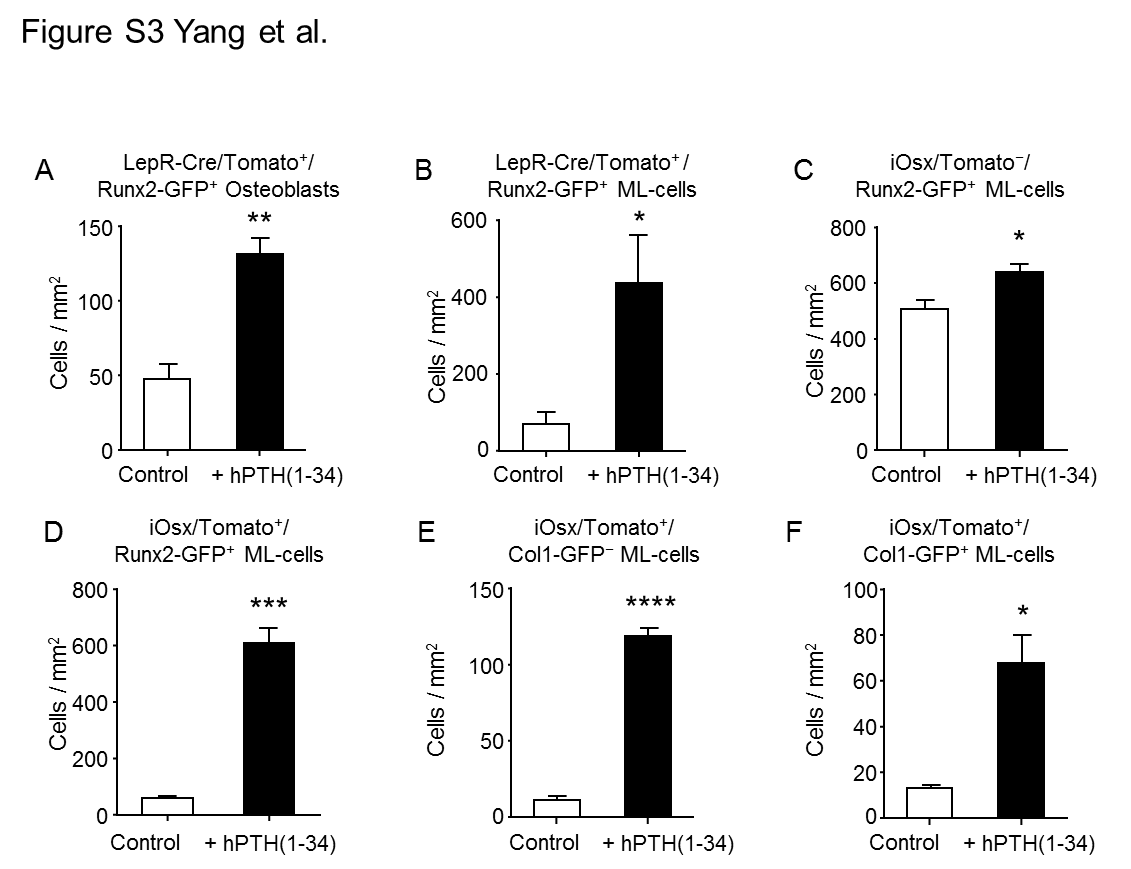
Figure S3. Osteoblastogenesis and multilayered (ML)-cells are induced in response to intermittent PTH treatment**

Six week-old LepR-Cre/Tomato/Runx2-GFP mice (A and B), tamoxifen-administered iOsx/Tomato/Runx2-GFP mice (C and D) and tamoxifen-administered iOsx/Tomato/Col1(2.3)-GFP mice (E and F) with vehicle and hPTH(1-34) intermittent treatment. Quantification of LepR-Cre/Tomato^+^/Runx2-GFP^+^ osteoblasts (A), LepR/Tomato^+^/Runx2-GFP^+^ ML-cells (B), iOsx/Tomato^−^Runx2-GFP^+^ ML-cells, (C), iOsx/Tomato^+^Runx2-GFP^+^ ML-cells (D), iOsx/Tomato^+^Col1(2.3)-GFP^−^ ML-cells (E) and iOsx/Tomato^+^Col1(2.3)-GFP^+^ ML-cells (F) in 1 mm^2^ within 200 μm from the bone surface. n=3-6 sections. **P<0.05, **P<0.01, ***P<0.001, ****P<0.0001.* Data are represented as mean ± SD.

**
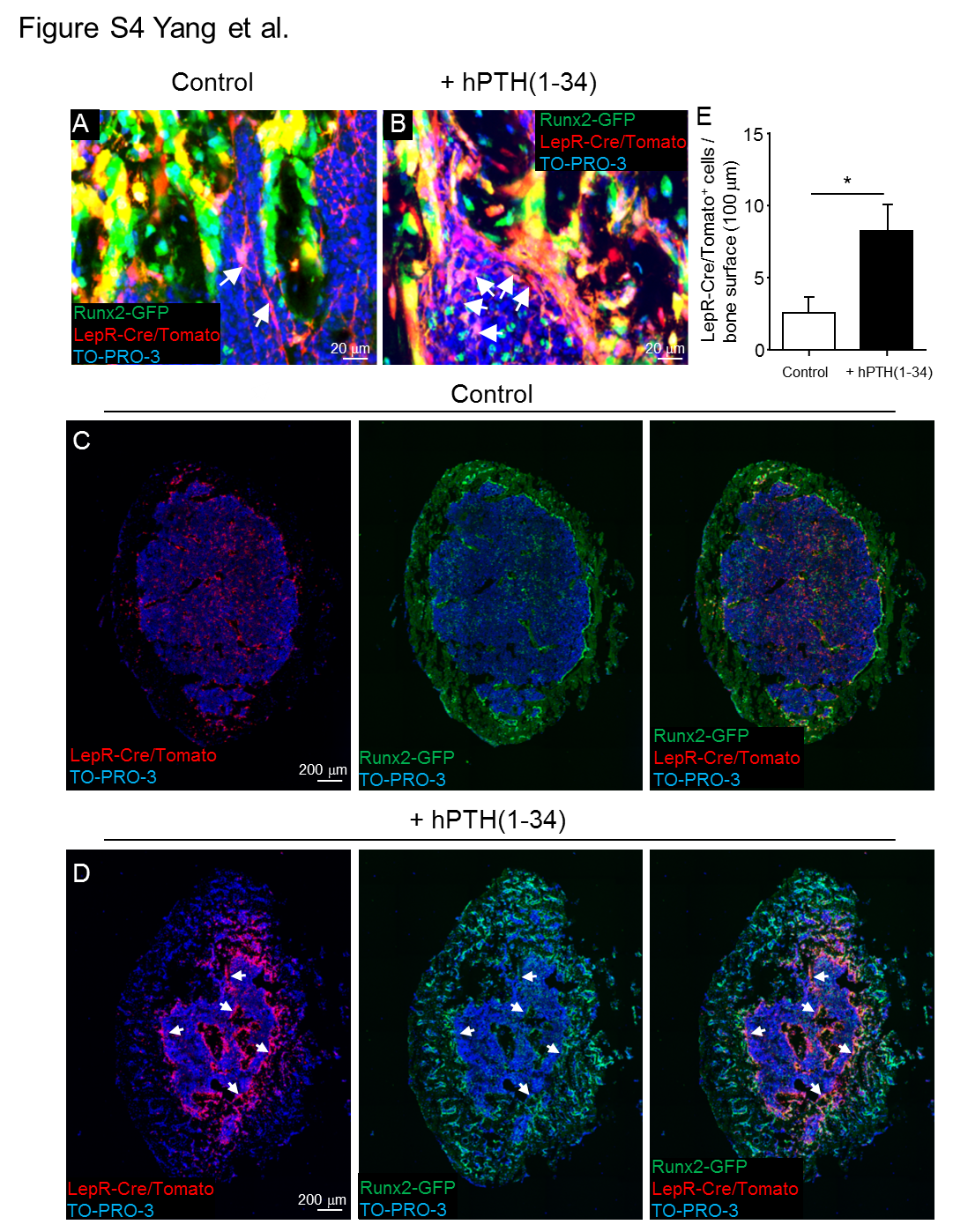
**

**Figure S4. Multilayered (ML)-cells are formed along the bone surface in response to intermittent PTH treatment.**

(A-D) Z-stack confocal images of thick bone sections of 6 week-old LepR-Cre/Tomato/Runx2-GFP mice with vehicle (A and C) and hPTH (1-34) (B and D) intermittent treatment. Arrows: LepR-Cre-derived Tomato^+^(LepR/Tomato^+^)Runx2-GFP^+^ cells (A, B and D). (E) Quantification of the number of LepR/Tomato^+^ ML-cells within 50 μm from the bone surface. n=3. **P<0.05*. Data are represented as mean ± SD. Nuclei were visualized with To-PRO-3 (blue).
